# Diversity of Coronaviruses with Particular Attention to the Interspecies Transmission of SARS-CoV-2

**DOI:** 10.3390/ani12030378

**Published:** 2022-02-04

**Authors:** Awad A. Shehata, Youssef A. Attia, Md. Tanvir Rahman, Shereen Basiouni, Hesham R. El-Seedi, Esam I. Azhar, Asmaa F. Khafaga, Hafez M. Hafez

**Affiliations:** 1 Birds and Rabbit Medicine Department, Faculty of Veterinary Medicine, University of Sadat City, Sadat City 32897, Egypt; Awad.Shehata@pernaturam.de; 2 Research and Development Section, PerNaturam GmbH, 56290 Gödenroth, Germany; 3 Department of Agriculture, Faculty of Environmental Sciences, King Abdulaziz University, P.O. Box 80208, Jeddah 21589, Saudi Arabia; yaattia@kau.edu.sa; 4 The Strategic Center to Kingdom Vision Realization, King Abdulaziz University, P.O. Box 80200, Jeddah 21589, Saudi Arabia; 5 Animal and Poultry Production Department, Faculty of Agriculture, Damanhour University, Damanhour 22516, Egypt; 6 Department of Microbiology and Hygiene, Faculty of Veterinary Science, Bangladesh Agricultural University, Mymensingh 2202, Bangladesh; tanvirahman@bau.edu.bd; 7 Clinical Pathology Department, Faculty of Veterinary Medicine, Benha University, Benha 13736, Egypt; shereenbh@yahoo.com; 8 School of Food and Biological Engineering, Jiangsu University, Zhenjiang 212013, China; hesham.el-seedi@farmbio.uu.se; 9 Department of Chemistry, Faculty of Science, Menoufia University, Shebin El-Kom 32512, Egypt; 10 Special Infectious Agents Unit—BSL3, King Fahd Medical Research Center and Department of Medical Laboratory Science, Faculty of Applied Medical Sciences, King Abdulaziz University, Jeddah 21362, Saudi Arabia; eazhar@kau.edu.sa; 11 Department of Pathology, Faculty of Veterinary Medicine, Alexandria University, Edfina 22758, Egypt; Asmaa.Khafaga@alexu.edu.eg; 12 Institute of Poultry Diseases, Faculty of Veterinary Medicine, Free University of Berlin, 14163 Berlin, Germany

**Keywords:** SARS-CoV-2, COVID-19, angiotensin-converting enzyme 2, interspecies transmission, receptors, One Health

## Abstract

**Simple Summary:**

Coronaviruses are a broad group of viruses that may infect a wide range of animals, including humans. Despite the fact that each coronavirus has a limited host range, frequent interspecies transmission of coronaviruses across diverse hosts has resulted in a complex ecology. The recently discovered SARS-CoV-2 virus is the clearest evidence of the danger of a global pandemic spreading. Natural infection with SARS-CoV-2 has been reported in a variety of domestic and wild animals, which may complicate the virus’s epidemiology and influence its development. In this review, we discussed the potential determinants of SARS-CoV-2 interspecies transmission. Additionally, despite the efforts that have been made to control this pandemic and to implement the One Health policy, several problems, such as the role of animals in SARS-CoV-2 evolution and the dynamics of interspecies transmission, are still unanswered.

**Abstract:**

In December 2019, the outbreak of severe acute respiratory syndrome coronavirus 2 (SARS-CoV-2) was reported in China with serious impacts on global health and economy that is still ongoing. Although interspecies transmission of coronaviruses is common and well documented, each coronavirus has a narrowly restricted host range. Coronaviruses utilize different receptors to mediate membrane fusion and replication in the cell cytoplasm. The interplay between the receptor-binding domain (RBD) of coronaviruses and their coevolution are determinants for host susceptibility. The recently emerged SARS-CoV-2 caused the coronavirus disease 2019 (COVID-19) pandemic and has also been reported in domestic and wild animals, raising the question about the responsibility of animals in virus evolution. Additionally, the COVID-19 pandemic might also substantially have an impact on animal production for a long time. In the present review, we discussed the diversity of coronaviruses in animals and thus the diversity of their receptors. Moreover, the determinants of the susceptibility of SARS-CoV-2 in several animals, with special reference to the current evidence of SARS-CoV-2 in animals, were highlighted. Finally, we shed light on the urgent demand for the implementation of the One Health concept as a collaborative global approach to mitigate the threat for both humans and animals.

## 1. Introduction

Since the first report of infectious bronchitis virus (IBV) in 1937 [1], numerous coronaviruses have been isolated and/or identified in various animal species as well as humans. The newly emerged severe acute respiratory syndrome coronavirus 2 (SARS-CoV-2) is the best example of a pandemic that had a global impact on the health, economic, and social aspects of communities [2,3]. At the time of writing this review, 350,292,303 SARS-CoV-2 cases and 5,611,459 deaths, according to Worldometer, had been reported worldwide. Although scientists have tried to control the coronavirus disease-19 (COVID-19) pandemic, the situation is still critical due to several global control strategy challenges. Like other coronaviruses, SARS-CoV-2 has acquired new mutations as a part of its evolution to evade host responses and transmit more effectively. Some of these mutations increased transmissibility through an increase in receptor binding or the ability to evade the host immune, in addition to the emergence of new variants, such as variant of interest (VOI) and variant of concern (VOC). The recently emerged “Omicron” variant (B.1.1.529) raises serious concern since it may significantly limit the antibody-mediated neutralization and increase the risk of reinfections due to the presence of numerous mutations in spike protein (30 nonsynonymous substitutions, 3 small deletions, and an insertion) [3].

Cross-species transmission events that force viruses to adapt to new host settings result in species-specific adaptations [4]. These evolutionary modifications may influence the virus’s virulence and transmissibility in new host species [5]. It is believed that the initial spillover of SARS-CoV-2 has a zoonotic transfer from bats to humans [6], possibly by an unidentified intermediate host(s); for instance, snakes, turtles, and pangolins have been proposed as the intermediate hosts [5,7]. Human-to-human transmission, the driving force behind the pandemic, has been confirmed even from asymptomatic carriers and presymptomatic infected persons [8,9]. Additionally, there is an incalculable number of daily human–animal interactions potentially leading to unreported zoonotic and anthroponotic transmission of SARS-CoV-2. The repeated interspecies virus transmission has the potential to speed up viral evolution and provide a source of new strain emergence. This review sheds light on the diversity of coronaviruses in domestic animals and the potential determinants of interspecies transmission of SARS-CoV-2.

## 2. Diversity of Coronaviruses in Domestic Animals

Coronaviruses belong to the family *Coronaviridae*, order *Nidovirales*. This family is classified into *Orthocoronavirinae* and *Letovirinae* subfamilies. According to the phylogenetic clustering, the subfamily *Letovirinae* includes only one genus (*Alphaletovirus*), while the *Orthocoronaviridae* subfamily consists of four genera: *Alphacoronavirus* (α-CoV), *Betacoronavirus* (β-CoV), *Gammacoronavirus* (γ-CoV), and *Deltacoronavirus* (δ-CoV). To date, a total of 17, 12, 2, and 7 species belonging to α-CoV, β-CoV, γ-CoV, and δ-CoV, respectively, are known and classified by the International Committee on Taxonomy of Viruses [10]. The diversity of coronaviruses in different animal species and humans is summarized in Table 1.

### 2.1. Avian Coronaviruses

According to the ICTV 2018, all γ-CoVs from any bird species are considered avian coronaviruses (ACoVs) regardless of the host, tropism, antigenicity, cross protection, and genome identity. The ACoVs includes several subspecies, such as IBV, guinea fowl coronavirus (GfCoV), turkey coronavirus (TCoV), duck coronavirus (DCoV), and pheasant coronavirus (PhCoV) [34,35]. However, this classification failed to establish a standard classification scheme that may collect all TCoVs into one genotypic relationship [36]. IBV, the first isolated coronavirus in the 1930s, is characterized by respiratory, intestinal, and urogenital problems in chickens [1,11]. In addition to respiratory complications, secondary infections with other microorganisms lead to a high mortality rate and a reduction of animal performance. Some strains can cause kidney failure, leading to fatal course. IBV can also duplicate in the oviduct, causing permanent impairment in young hens. The primary host of IBV is chicken (*Gallus gallus*), but the virus has also been reported in pheasant and peafowl [37]. It was proposed that TCoV is emerged because of a recombination that occurred between IBVs and an unknown coronavirus. These recombinations and mutations lead to interspecies transmission from chickens to turkeys and change the virus tropism from respiratory to enteric [37,38]. TCoV is intended to be involved in the poult enteritis and mortality syndrome (PEMS) in turkeys [36]. The emergence of IBV variant strain β-CoVs, caused by the high mutation rate, makes the control difficult.

### 2.2. Coronaviruses in Pigs

To date, six coronaviruses are known in pigs, belonging to α-CoVs, β-CoVs, and δ-CoVs. Among the α-CoVs that can infect pigs are transmissible gastroenteritis virus (TGEV), porcine epidemic diarrhea virus (PEDV), porcine respiratory coronavirus (PRCV), and swine acute diarrhea syndrome coronavirus (SADS-CoV). The porcine hemagglutinating encephalomyelitis virus (PHEV) belongs to β-CoVs, and porcine deltacoronavirus (PDCoV) belongs to δ-CoVs, which are also known to infect pigs [16,17,18,19,20,21].

### 2.3. Coronaviruses in Dogs

Two canine coronaviruses in dogs are known: canine coronavirus (CCoV) and canine respiratory coronavirus (CRCoV). CCoV belongs to α-CoV and causes acute enteritis in young dogs (1- to 12-week-olds), where sometimes the infection is subclinical. The CCoV-β-CoV infection is sometimes accompanied with respiratory manifestation. Based on the spike gene, two distinct serotypes of CCoV are known: CCoV-I and CCoV-II. CCoV-II is subdivided into CCoV-IIa and CCoV-IIb, in which CCoV-IIb is a recombinant virus between CCoV-II and TGE [38,39]. On the other hand, CRCoV belongs to β-CoV, causes respiratory manifestations in dogs older than 2 years, and is the key player of canine infectious respiratory disease development (CRID), or “kennel cough” syndrome [22,23,40,41,42].

### 2.4. Coronaviruses in Cats

Feline coronavirus (FCoV) belongs to α-CoVs and causes disease in domestic and nondomestic felid species. The infection is associated with mild to severe immune-mediated disease (feline infectious peritonitis or FIP) [24,43].

### 2.5. Coronaviruses in Cattle

In cattle (*Bos taurus*), bovine coronavirus (BCoV) belongs to β-CoV and causes economic losses due to respiratory and gut problems [44]. In calves, BCoV causes severe bloody diarrhea due to the destruction of intestinal villi, resulting in high mortality rates [45]. In adult cattle, it also induces severe or fatal infection, especially in case of coinfections with other secondary respiratory pathogens [45,46,47].

### 2.6. Coronaviruses in Equines

Equine coronavirus (ECoV) belongs to β-CoV and was first isolated from the feces of a diarrheic foal in 1999 in North Carolina, USA [26]. In the last decade, it has been associated with outbreaks of enteric disease in adult horses in the USA, Europe, and Japan [48,49,50,51,52,53]. The virus was detected also in other several countries, including Saudi Arabia and Oman [54]. Recently, ECoV antibodies were detected in horses in Israel [55].

### 2.7. Coronaviruses in Humans

**Figure 1:**
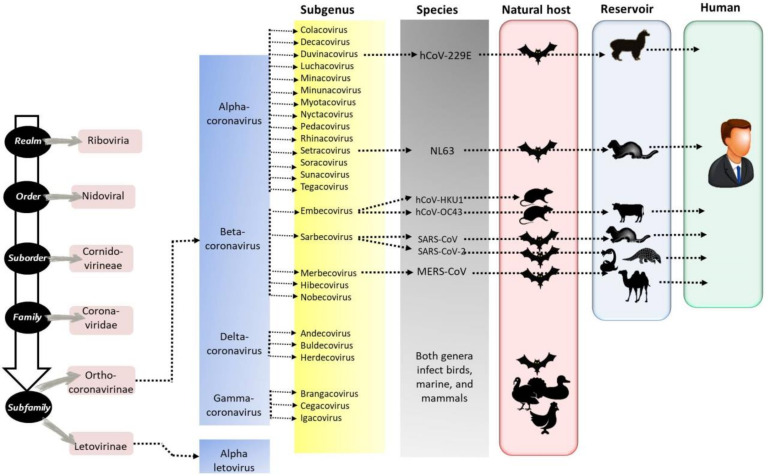
Diversity of coronaviruses. Seven human coronaviruses are known, explicitly, 229E, NL63 (α-CoVs), HKU1, OC43, severe acute respiratory syndrome (SARS-CoV), SARS-CoV-2 (β-CoVs), and Middle East respiratory syndrome coronavirus (MERS-CoV), adapted from the International Committee on Taxonomy of Viruses [10].

To date, seven coronaviruses are known to be able to infect humans, explicitly, 229E, NL63 (α-CoVs), OC43, HKU1, Middle East respiratory syndrome coronavirus (MERS), severe acute respiratory syndrome (SARS), and SARS-CoV-2 (β-CoVs) (Figure 1). NL63, 229E, OC43, and HKU1 cause only mild respiratory diseases and enteritis, similar to the common cold [56]. However, MERS-CoV and SARS-CoV-2 cause acute fatal pneumonia [33,57,58]. Three coronavirus pandemics/epidemics were reported in humans [47]: (i) SARS-CoV epidemic reported in Asia in 2002 (8422 cases and 11% mortality rate), (ii) MERS-CoV-2 reported in Saudi Arabia in 2012 (2468 cases, 34% mortality rate), (iii) the newly emerged SARS-CoV-2 in 2019 (~2% mortality rate).

## 3. Diversity of Coronavirus Receptors

Coronaviruses utilize different receptors to mediate membrane fusion and to enter the host cell and replicate in the cytoplasm. Therefore, interspecies transmission could be ascribed to the large S-glycoprotein. The S-protein homodimers consist of two subunits: S1 subunit (accountable for binding with a receptor) and S2 subunit (mediated membrane fusion). The S1 subunit is subdivided into the N-terminal domain (NTD) and the C-terminal domain (CTD). On the other hand, the S2 subunit is subdivided into a fusion protein (FP) that plays a role in membrane fusion, heptad repeat 1 (HR1) and HR2. Either NTD or CTD domains can bind to distinct receptors and serve as the receptor-binding domain (RBD) [58]. However, the RBD of coronaviruses is located within the S1 subdomain of the spike protein, which might allow binding to different receptors [59]. For example, although the receptor-binding domain (RBD) of most coronaviruses is located in the CT, the RBD of murine hepatitis virus (MHV-A59) is located within the NTD of the S-protein [60,61,62,63,64,65]. Additionally, although the RBD is relatively well conserved, the receptor-binding motif (RBM) tends to mutate frequently and determines receptor specificity [64]. In this section, we will shed light on the different receptors of coronaviruses. The receptors that are utilized by coronaviruses are shown in Table 2.

### 3.1. Amino Peptidase Receptors

Aminopeptidase N receptors (APNs, CD13), a type II transmembrane zinc aminopeptidase and protein, are utilized by several species of the α-CoV genus, such as HCoV-229E, TGEV, PRCV, FCoV, and CCoV [66,67,69,80]. The APN is found in the gut, nervous system, dendritic cells, and monocytes of various hosts [81,82]. Coronaviruses that bind to the APN use their S1 CTD domain. However, the mode of interaction between the RBD and APN is distinct in different α-CoVs [83]. In [84], it was found that SARS-CoV-2 is unable to bind to the APN receptors.

### 3.2. Carcinoembryonic Antigen-Related Cell Adhesion Molecule 1

Carcinoembryonic antigen-related cell adhesion molecule 1 (CEACAM1) belongs to the immunoglobulin superfamily. As a cellular adhesion molecule, CEACAM1 is utilized by the mouse hepatitis virus (MHV) and belongs to β-CoV [85].

### 3.3. Dipeptidyl Peptidase 4 (DPP4)

The dipeptidyl peptidase 4 (DPP4) is a serine exopeptidase type II transmembrane protein. It is utilized by several coronaviruses such as MERS-CoV, camel-derived MERS-CoV, and BatCoV HKU4 [73]. The DPP4 receptors are expressed ubiquitously in the lungs, liver, intestine, immune cells, and kidneys [86]. The DPP4 receptor might be involved in SARS-CoV-2 infection [84]; however, their exact role in SARS-CoV-2 pathogenesis needs further investigation.

### 3.4. Sialic Acids or Sialosides, Acidic Carbohydrates

Like other viruses, such as parainfluenza, rotaviruses, adenoviruses, polyomaviruses, and influenza viruses [87,88], some coronaviruses can also bind to sialic acids or sialosides and acidic carbohydrates [73,75,78,79] (Table 2). Both HCoV-OC43 and BCoV bind to the N-acetyl-9-*O*-acetylneuraminic acid to mediate cellular entry [89,90,91,92]. Additionally, in [92] it was also suggested that the S-protein of SARS-CoV-2 might binds to α, N-acetyl neuraminic acid. This interaction needs to be investigated further because it might have a role in enteric tract infections.

### 3.5. Angiotensin-Converting Enzyme 2 (ACE2)

**Figure 2:**
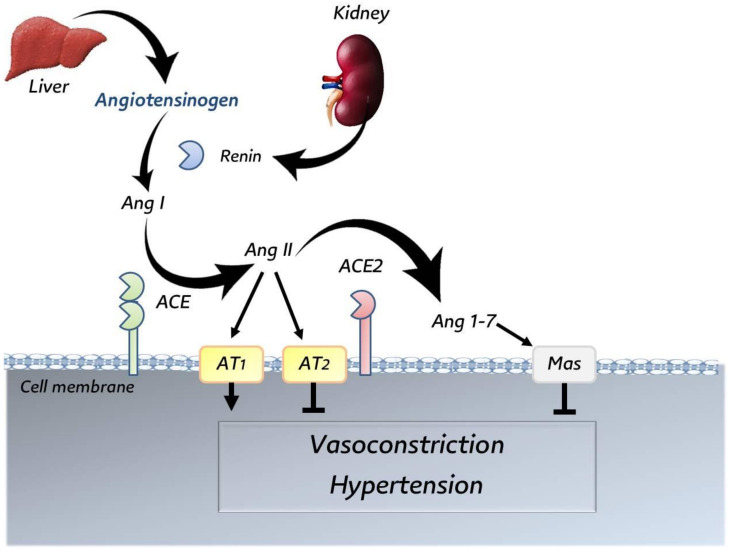
Catalytic function of ACE2 in renin–angiotensin system (RAS). Adapted after Bader et al. [98].

ACE2 is used by several coronaviruses, namely, SARS-CoV [68], NL63 [71], and SARS-CoV-2 [72]. The high sequence similarities between the receptor-binding domains (RBDs) of these viruses [92] might explain why these viruses interfere with the same receptor. The ACE2 receptor was discovered in 2000s. It is widely expressed in different organs and tissues, including pulmonary and extrapulmonary [93,94,95], and has specific functions, much more than just a receptor for SARS-COV-2: (i) catalytic function of the renin–angiotensin system (RAS); hence, it regulates and maintains the relative stability of the body homeostasis and the balance of blood pressure and water and electrolytes [96,97,98]. ACE2 has a catalytic function in RAS, which is summarized in Figure 2. (ii) ACE2 has a significant role in the transport of amino acids. (iii) ACE2 is abused by several viruses (i.e., SARS-CoV, SARS-CoV-2, and NL63) as a receptor to promote virus entry. The RBD of SARS-CoV-2 binds to the peptidase domain (PD) of ACE2, which is critical for viral entry [95,98].

## 4. Interaction between SARS-CoV-2 and ACE2 Receptor

**Figure 3:**
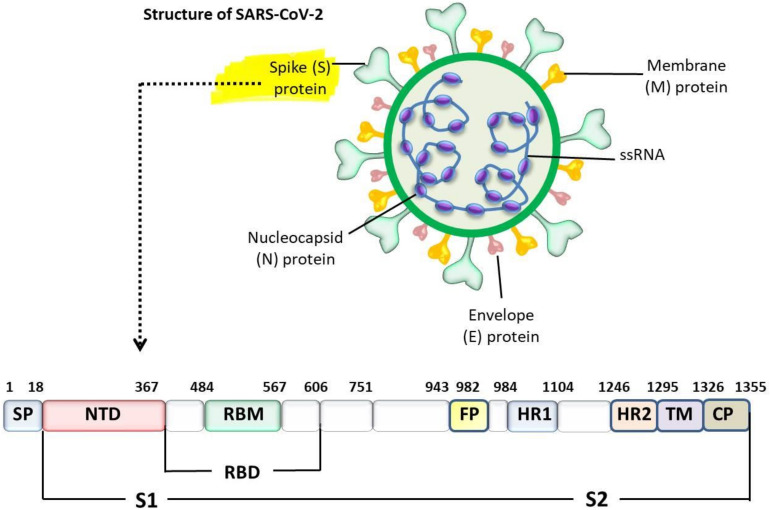
The general organization of the coronavirus S-protein. SP = signal peptide, NTD = N-terminal domain, CTD = C-terminal domain, RBD = receptor-binding domain, RBM = receptor-binding motif, FP = fusion peptide, HR1 = heptad repeat 1, HR2 = heptad repeat 2, TM = transmembrane domain (TM), CD = C-domain.

The entry of SARS-CoV-2 into the cells correlates with the binding of the RBD of the S1 subunit to ACE2 and the priming by host cell proteases, which cleave the S-protein at the S/S2 and subsequently allow cellular membrane fusion and cell entry [72,84,92,99,100]. The general organization of the coronavirus S-protein is shown in Figure 3. In [101,102], it was found that transmembrane serine protease 2 (TMPRSS2) can cleave the S-glycoprotein at the S2′ site and furin proteases at the S1/S2 site. There are four sequential steps for the cell entry of coronaviruses: (i) cleavage of S1/S2 by the proprotein convertase furin, which cleaves at R-X-R/K-R↓. Furin is a ubiquitous protease expressed in eukaryotic cells. It has physiological functions in transport secretory pathways via the cleavage of several proteins, such as cell surface receptors, hormones, adhesion molecules, and growth factors [103]. Unfortunately, this enzyme is abused by several viruses, such as avian influenza, Ebola, yellow fever, human immunodeficiency virus, measles, and bacterial toxins, to facilitate cell entry [104]. The cleavage of the S1/S2 site might induce conformational changes that might be required for receptor binding [103,105,106]. The R-R-A-R motif of SARS-CoV-2 at the junction between the S1 and S2 subdomains is sensitive to furin cleavage [72]. (ii) SARS-CoV-2 binds with ACE2 receptors. (iii) The cleavage of the S-protein of SARS-CoV-2 by TMPRSS2 at single arginine or lysine residues (R/K↓), which, subsequently, allow internalization. (iv) Membrane fusion and virus entry are achieved by direct fusion with the plasma membrane or use of endocytic mechanisms.

## 5. Interspecies Transmission of Coronaviruses

**Figure 4:**
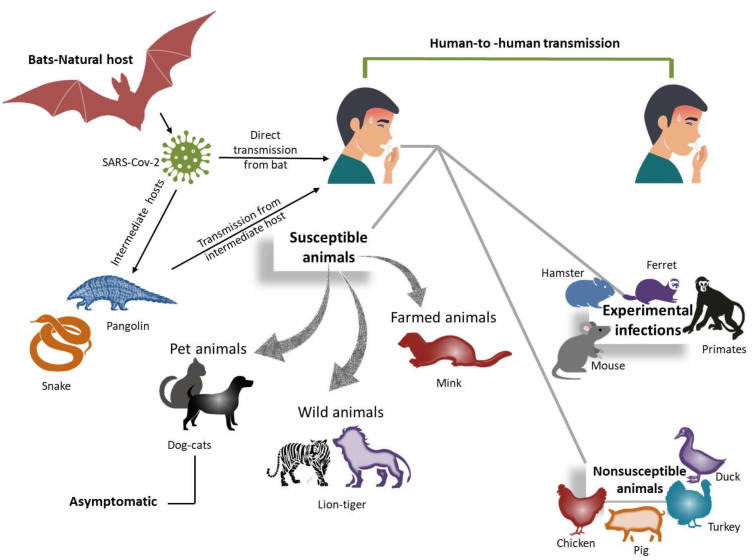
Susceptibility of different animals to SARS-CoV-2.

Bats (*Rhinolophus affinis*) are a reservoir for several human coronaviruses, such as NL63, 229E, MERS-CoV, and SARS-CoV [107,108,109,110,111,112]. Rodents are the natural host of both human CoVs OC43 and HKU1 [113]. Although the intermediate hosts of both HKU1 and NL63 are unknown, bovines, camelids, dromedary camels (*Camelus dromedarius*), and palm civets (*Paradoxurus hermaphroditus*) are the intermediate hosts to OC43, 229E, MERS-CoV, and SARS-CoV, respectively [57,107,108,109,110]. Recently, a novel canine–feline recombinant virus was found in a human pneumonia patient [111]. Generally, coronaviruses are observed to cross “species barriers” easily for several reasons, including: (i) The high mutation rates [112], site-directed mutations targeting the S-gene of the MHV to replace the ectodomain of its S-protein with the highly divergent ectodomain FIPV, resulting in the ability to infect feline cells and simultaneously missing the capacity to infect murine cells [113]. Moreover, the manipulation of the FIPV genome leads to switching species tropism [114]. Additionally, interspecies switching of IBV, cell tropism of FECoV, and FIPV highlight that the S-glycoprotein is a determinant of cell tropism. (ii) The large RNA (~29,903 nucleotides) genome of the coronaviruses raises the commonness of recombination and mutation events, resulting in the development of new strains/variants [115]; (iii) Coevolution between coronaviruses and receptors was cited by Bolles and others [116]. Although coronaviruses utilize specific cell receptors for cell entry, the S-protein binds with receptors and subsequently activates fusion; coevolution between virus (S-protein) and receptors is common, and hence, it can extend or alter the host range and jump the “species barrier”. For example, the RBD of SARS-CoV isolated in 2002–2003 demonstrated a greater binding affinity to ACE2 than that isolated in 2003–2004. Indeed, both receptors and coronaviruses are flexible and undergo mutations that lead to conformational changes required for host adaptation. Accumulation of the mutations of the S-protein is necessary for host adaptation [117,118]. The following section discusses the susceptibility of various animal species to the newly emerged SARS-CoV-2 and the determinants of different susceptibilities. Figure 4 illustrates the exposure of several animals to SARS-CoV-2.

### 5.1. SARS-CoV-2 in Dogs and Cats

Several studies have reported the detection of SARS-CoV-2 in pet animals, such as dogs (*Canis lupus familiaris*) and cats (*Felis catus*). In Hong Kong, SARS-CoV-2 was detected in asymptomatic Pomeranian and German shepherd dogs in which the owners also tested COVID-19 positive. The sequence of these viruses exhibited high identities with the respective human cases, highlighting the potential human–animal transmission [119,120]. Additionally, neutralizing antibodies against SARS-CoV-2 were also identified using plaque reduction neutralization assays. In Italy, extensive research was conducted to assess SARS-CoV-2 infection in companion animals (n = 999), sampled at a time of frequent human disease. Although all samples were negative using PCR, neutralizing antibodies against SARS-CoV-2 were recorded in dogs (3.3%) and cats (5.8%). The incidence of positive cases was significantly higher in dogs maintained in COVID-19-positive households than those maintained in COVID-19-negative households [120]. In Brazil, SARS-CoV-2 was also identified in dogs and cats that kept close to COVID-19-positive human cases [121]. In France, Fritz and others also found that 58.8% (20/34) of tested cats and 38.5% (5/13) of tested dogs were seropositive for SARS-CoV-2 [122]; positive samples were greater among animals kept close to COVID-19-positive households. In Lima, Peru, SARS-CoV-2 antibodies were also observed in domestic cats [123]. In Germany, during the first-wave COVID-19 pandemic, antibodies to SARS-CoV-2 were detected in 0.65% (6/920) of serum samples collected from cats [124]. Later on, during the second wave (between September 2020 and February 2021), the prevalence of positive samples was significantly higher. Out of 1173, a total of 16 samples were positive (1.36%) [124]. In the Netherlands, SARS-CoV-2 was detected in pets kept in an infected mink farm using PCR and serological examination. Although no cat-to-cat infection of SARS-CoV-2 was observed in this study, mink-to-cat transmission was documented, highlighting the role of mink as a potential threat for disseminating the virus [125]. The UK B.1.1.7 variant of SARS-CoV-2 was detected for the first time in a cat and a dog in the US. After their homeowners were diagnosed with COVID-19, the animals tested positive 2 days later [126]. Both animals exhibited respiratory manifestations several weeks after virus detection. In the United Kingdom, the B.1.1.7 variant was also detected in a dog and 2 cats whose owners were also COVID-19 positive [127]. These animals exhibited no respiratory signs but showed cardiac abnormalities (myocarditis). Additionally, SARS-CoV-2 was investigated in feral cats near mink farms diagnosed to be COVID-19 positive in the Netherlands. Seven out of 24 tested animals had antibodies to SARS-CoV-2, and 1 cat was confirmed positive for virus RNA [128].

Based on the documented report, we can conclude the following: (i) Dogs that tested positive for SARS-CoV-2 did not exhibit any clinical symptoms [121], with the exception of transient respiratory distress and lethargy found in only one case [129]. (ii) Cats are more sensitive to SARS-CoV-2. Most SARS-CoV-2-positive cats exhibited diarrhea, vomiting, and respiratory manifestations [129]. These clinical findings are in accordance with the predicting susceptibility studies of Alexander et al. [130]. Additionally, although both dogs and cats have ACE2 receptors in the respiratory tract, dogs harbor low levels of ACE2 compared with cats [131,132]. This might explain why cats are more susceptible than dogs [133]. (iii) Cross-reactivity between coronavirus antibodies is possible, which must be considered in serological assays [134,135,136]. For example, SARS-CoV-2 exhibited a homology of 45% with CCoV. However, β-CoV, HCoV-OC43, and CRCoV exhibited a homology of 97% [137]. (iv) Although anthropozoonotic transmission (human to animal) of SARS-CoV-2 is possible, to date, there is a low risk of zoonotic transmission (animal to human) [137,138,139,140,141,142]. (v) The available data are not sufficient to rule out cat-to-cat transmission. Further investigations are required.

### 5.2. SARS-CoV-2 in Mink

Two different minks, the family *Mustelidae*, are known, namely, European (*Mustela lutreola*) and American (*Neovison vison*) mink. Minks are aggressive animals and thus not suitable as pets. However, American minks are used worldwide for production [143]; Poland, the Netherlands, Denmark, and China are the main producing countries. Furthermore, in several countries in Europe and the Americas, SARS-CoV-2 has been found among minks (*Neovison vison*), suggesting that mink might be a reservoir of SARS-CoV-2 [128,144].

In May 2020, SARS-CoV-2 was reported in mink in Denmark for the first time [145]; then the virus was also documented in the Netherlands in mid-June 2020. Due to the possible zoonotic transmission of the virus, a culling strategy of mink farms was implemented [146]. In the US, the virus was reported in farmed mink on 17 August 2020. The virus was also detected in Italy, France, Sweden, Spain, Poland, Canada, Greece, and Lithuania. However, Russia and China have not seen SARS-CoV-2 in their minks [143].

Generally, SARS-CoV-2 multiplies effectively in the respiratory system of mink, leading to respiratory lesions like COVID-19 in humans. It was also cited that an S-protein-based vaccine in mink farms prevented the SARS-CoV-2 replication and lesions induced by SARS-CoV-2. This highlights the potential of mink as a valuable animal model for studying the pathogenesis of SARS-CoV-2 prior to the evaluation of an antiviral drug and vaccines [147].

Considering the epidemiology and clinical picture of SARS-CoV-2 in farmed minks, the following points can be concluded: (i) The detection of SARS-CoV-2 in farmed mink demonstrated that mink might be an intermediate host and played a prospective role in the early phases of the pandemic transmission [143]. (ii) It also remains to be investigated whether mink has an impact on the virus evolution due to its rapid spread as a new host, which might lead to the emergence of mutations. It was found that SARS-CoV-2 evolved slower in humans than in minks [148] due to adaptation of the virus under selective pressure. Thus, continuous tracing of the molecular changes of SARS-CoV-2 in mink is essential to explore virus evolution.

### 5.3. SARS-CoV-2 in Rabbits

Rabbits (*Oryctolagus cuniculus*) are vulnerable to SARS-CoV-2 [149]. After tentative infection of rabbits with SARS-CoV-2, the virus was detected in nasal swabs for 7 days postinoculation with a peak titer of 10^3^ TCID_50_. However, the disease was asymptomatic [150]. Therefore, further surveys are needed to identify the prospective role of rabbits in SARS-CoV-2 evolution.

### 5.4. SARS-CoV-2 in Other Animals

The first records of natural SARS-CoV-2 infection in lions (*Panthera leo*) and tigers (*Panthera tigris*) were reported in the US by McAloose and others (Table 3) [151]. The virus was noticed in feces and respiratory secretions. Most of the symptoms were related to the respiratory system, particularly cough. In Croatia, jackals (*Canis aureus*), free-living wild boars (*Sus scrofa*), and red foxes (*Vulpes vulpes*) showed positive antibody response to SARS-CoV-2 using ELISA. However, the virus neutralization test’s positive conclusions were not verified [152]. However, chickens, geese, and pigs were found to be nonsusceptible hosts for SARS-CoV-2 [153,154,155]. On the other hand, 8-week vintage crossbred pigs (*Sus scrofa domesticus*) were observed to be vulnerable to SARS-CoV-2 infection subsequent to oronasal infection. Although RNA from SARS-CoV-2 has been detected in nasal and oral fluids from pigs, the live virus has only been detected in the tissues of one animal. Moreover, SARS-CoV-2 neutralizing antibodies were found postinfection in oral fluid and serum in [156]. Experimental studies produced contradictory results since the infected animals were of different ages and breeds. In addition, they used different viral isolates and infectious doses [153,154,155,156]. Pickering and coworkers [156] applied an increasing viral level (10-fold) for investigational inoculation compared with previous experiments. In vitro, SARS-CoV-2 can replicate and induce cytopathic effects in the porcine kidney (PK-15) and swine testicle (ST) cell lines. Nevertheless, experimental inoculation of 5-week-old pigs did not show clinical signs in [153]. Additional investigations are needed to validate the exposure of pigs to disease.

Although an ACE2 receptor is present in almost all animals, there are different susceptibilities to SARS-CoV-2. Therefore, considering the predicting susceptibility studies, animals can be categorized into four groups [84,119,130,155,157,158,159,160]. (i) Susceptible animals such as cats (*Felis catus*), tigers (*Panthera tigris altaica*), lions (*Panthera leo*), rhesus macaques (*Macaca mulatta*), and golden Syrian hamsters (*Mesocricetus auratus*). (ii) Intermediate susceptible animals such as pigs (*Sus scrofa*), ferrets (*Mustela putorius furo*), and dogs (*Canis lupus familiaris*). (iii) Nonsusceptible animals including chickens (*Gallus gallus*), ducks (*Aythya fuligula*), geese (*Anser cygnoides*), Japanese quails (*Coturnix japonica*), and mice (*Mus musculus*). (iv) Unknown susceptible animals were recorded so far, such as camels (*Camelus bactrianus* and Arabian camel), horses (*Equus caballus*), Malayan pangolins *(Manis javanica*), and sheep (*Ovis aries*).

Generally, the binding of the SARS-CoV-2 S-glycoprotein to ACE2 is essential for virus duplication. However, the overall homology of ACE2 among animals is not the determinant for SARS-CoV-2 infection for the two following reasons: (i) ACE2 of mouse (not susceptible) is more similar to that of golden Syrian hamster (*Mesocricetus auratus*, susceptible animal) than ducks and chickens (nonsusceptible) [84,161]. (ii) Considering the phylogenetic analysis, mice are more similar to humans and rhesus macaques (susceptible species) than ducks and chickens (nonsusceptible species) [84,161].

Indeed, there are some potential determinants for the host specificity and pathogenicity of SARS-CoV-2: (i) It is suggested that ACE2 amino acid residues, namely, 30, 83, 90, 322, and 354, can be used to differentiate between susceptible and nonsusceptible hosts. Alexander and coworkers [130] established a susceptibility score for SARS-CoV-2 infection in several animals, in which the lower cut-off for susceptibility is 23 and the upper cut-off for nonsusceptibility is 11. (ii) Coevolution between ACE2 receptors and the RBD of SARS-CoV-2 can lead to potential adaptation. Both ACE2 and SARS-CoV-2 are flexible and prone to mutations that affect the binding affinity to an ACE2 receptor, which defines the pathogenicity of the virus [61] and may lead to its adaptation in a new host. Several amino acid residues of the ACE2 receptor interface with certain residues of the SARS-CoV-2 RBD, namely, Y453F, F486L, and N501T residues, differ between humans and minks [162]. It is suggested that these differences may underlie the selective pressure for SARS-CoV-2 to adapt to a mink ACE2 receptor. It was found that the amino acid mutations Y453F, F486L, and N501T provide a better interaction between ACE2 and SARS-CoV-2 [98].

**Table 3:** Susceptibility of animals to SARS-CoV-2 virus under experimental and natural infections.

Risk Level	Animals	Experimental	Natural	Remarks	References
Low	Dog (*Canis lupus familiaris*)	+	+	No symptoms	[129]
	Cattle (*Bos taurus*)	+	-	No symptoms	[163]
	Common marmosets (*Callithrix jacchus*)	+	+	No symptoms	[164]
	Tree shrew (*Tupaia belangeri*)	+	+	No symptoms	[95]
High	Cat (*Felis catus*)	+	+	mild symptoms	[155,165]
	Malayan tiger (*Panthera tigris* subsp. *jacksoni*)	+	+	Symptoms	[63]
	Lion (*Panthera leo*)	-	+	Symptoms	[151]
	Puma (*Puma concolor*)	-	+	Symptoms	[165]
	American mink (*Neovison vison*)	-	+	Symptoms	[128,148]
	Egyptian fruit bats (*Rousettus aegyptiacus*)	+	-	No symptoms	[154]
	Ferret (*Mustela putorius furo)*	+	-	Very mild	[155,157,166]
Detected	Rabbits (*Oryctolagus cuniculus*)	+	-	No symptoms	[149]
	Raccoon dogs (*Nyctereutes procyonoides*)	+	-	No symptoms	[167]
	North American raccoons (*Procyon lotor*)	+	-	No symptoms	[168]
	Striped skunks (*Mephitis mephitis*)	+	-	No symptoms	[168]
	White Chinese geese (*Anser cygnoides*)	+	-	No symptoms	[169]
Nonsusceptible	Japanese quail (*Coturnix japonica*)	+	-	No symptoms	[170]
	White Chinese geese (*Anser cygnoides*)	+	-	No symptoms	[170]
	Turkeys (*Meleagris gallopavo*)	+	-	No symptoms	[170]
	Pekin duck (*Anas platyrhinchos domesticus*)	+	-	No symptoms	[170]
	Duck (*Anatidae*)	+	-	No symptoms	[155]
	Equine (*Equus caballus*)	+	-	No symptoms	[171]

## 6. Control Measures and Implementation of One Health Strategy

The control of the SARS-CoV-2 pandemic still demands the One Health concept as a collaborative global approach to mitigate risk for both humans and animals (domestic and wildlife) and unravel and prevent the seriousness of complex a human–animal environmental health problem. To implement the One Health concept, all relevant stakeholders, including physicians and public health experts, veterinarians, epidemiologists, diagnosticians, pharmaceutical companies, vaccinologists, governments, and economists, must be engaged to identify clinical cases, perform laboratory diagnosis, trace the virus epidemiology, control the disease, enhance the isolation, quarantine, cure, vaccinate humans, and initially raise public awareness. In this regard, the following measures are described by several authors [172,173,174]: (i) developing strategies and funding needed for the application of preventative and control measures in the frame of One Health, (ii) engagement of well-trained and professional staff, (iii) fast and precise diagnostic tools and treatment of affected individuals, (iv) development and provision of efficient and safe vaccines for humans, (v) biosurveillance of live animal markets and humans in contact with animals to identity the possible reservoirs and to assess the risk factors, (vi) application of biosecurity in animal farms and implementation of good hygienic measures, (vii) assessment of the economic and social impacts of COVID-19 on people, and (viii) provision of efficient drugs and vaccines against SARS-CoV-2 and diagnostics. To control the spread of zoonotic diseases, vaccinating animals is more cost-effective, as well as (ix) cooperation among different agencies and taking advantage of veterinarians and human medicine experiences in the virus isolation programs and in the disinfection and collection of premises and clinics under the supervision of health authorities in order to prevent and/or reduce human outbreaks, (x) raising public awareness of SARS-CoV-2 transmission, and (xi) providing safe work practices in healthcare and nonhealthcare workplaces, and it is recommended to reassess the risk of COVID-19 infection from time to time. (xii) Protecting forests and changing agricultural practices are essential and cost-effective actions to prevent pandemics. Thus, destruction of the ecosystem by anthropogenic actions (such as urbanization, agricultural expansion, deforestation, and globalization) could be the reason for the emergence of pandemics. Unfortunately, it is widely highlighted that people became infected with SARS-CoV-2 through interaction with wild animals at the Huanan seafood wholesale market [175]. (xiii) As animal-to-human transmission of SARS-CoV-2 is not ruled out, the concept of One Health is urgently needed to control this pandemic virus. (xiv) Cleanliness and environmental hygiene are also important. Interaction with animals and improper use of animal products during COVID-19 outbreak must be avoided.

## 7. Conclusions and Recommendations

Coronaviruses are diverse and can infect a wide range of animal species as well as humans. Currently, coronaviruses are categorized into four genera, specifically, αCoV, βCoV, γ-CoV, and δ-CoV. Although each coronavirus has a narrow-restricted host range, the frequent interspecies transmission of coronaviruses between different hosts leads to a complex ecosystem. The newly emerged SARS-CoV-2 is the clearest example of the risk of the spread of a disastrous pandemic worldwide. Natural infection with SARS-CoV-2 has been stated in several domestic and wild animals—dogs, cats, mink, ferrets, lions, tigers, pumas, snow leopards, and gorillas—which might contribute to complicating the epidemiology of the virus and have an impact on virus evolution. Experimentally, dogs, ferrets, cats, rhesus macaques, cynomolgus macaques, rabbits, white-tailed deer, and Syrian hamsters are susceptible to SARS-CoV-2 infections. Both humans and some animals have encountered unfavorable healthy problems due to SARS-CoV-2 infection. Therefore, while certain animals are resistant to SARS-CoV-2, adequate management, and strict hygienic processing of animal products should be considered during marketing and handling as a general rule for all animal supplies. On the other hand, the determinants of host susceptibility need further investigations to point out the factors that contribute to interspecies transmission. Although authorities, scientists, and industries have made several efforts to control this pandemic and have implemented the One Health strategy, several unresolved questions still pose significant challenges to researchers, such as: (i) What is the intermediate host of SARS-CoV-2? (ii) What is the role of animals in the early animal-to-human infection and in virus evolution? (iii) What are the dynamics and determinants of the interspecies transmission of this virus?

A future pandemic could be worse than the ongoing COVID-19 because we are pushing nature to its limits by destroying and degrading amazing diverse ecosystems and ultimately removing natural buffers and expanding the interface between wildlife and people where pandemics emerge. Therefore, a multidisciplinary One Health task must be implemented to avoid the emergence of new pandemics. This is a call for scientists to explore in more detail the role of animals in this pandemic, maybe through experimental studies, expanding the ongoing surveillance and molecular epidemiology of SARS-CoV-2 to relevant animal populations. In addition, there is an urgent demand to implement the One Health concept to combat the COVID-19 pandemic with a holistic approach.

## Figures and Tables

**Table 1:** Diversity of coronaviruses in different animal species and humans.

Host	Virus *	Genus	First Year of Isolation	Country of First Isolation	Tropism	References
Avian species	IBV	β-CoVs	1930	USA	Respiratory, urinary, and reproductive	[1,11]
	TcoV	β-CoVs	1971	USA	Enteric	[12]
	PhCoV	β-CoVs	1980	UK	Respiratory, reproductive, urinary	[13]
	GfCoV	β-CoVs	2011	France	Fulminating enteritis	[14]
	PiCoV	β-CoVs	1988	Australia	Enteric	[15]
Pig (*Sus scrofa domesticus*)	TGEV	α-CoVs	1946	USA	Enteric	[16]
	PEDV	α-CoVs	1971	UK	Enteric	[17]
	PRCV	α-CoVs	1986	Belgium	Respiratory	[18]
	SADS-CoV	α-CoVs	2017	China	Enteric	[19]
	PHEV	β-CoVs	1962	Canada	Respiratory, nervous	[20]
	PDCoV	δ-CoVs	2012	China	Enteric	[21]
Dog (*Canis lupus familiaris*)	CCoV	α-CoVs	1971	Germany	Acute enteritis	[22]
	CRCoV	β-CoVs	2003	UK	Respiratory	[23]
Cat (*Felis catus*)	FCoV	α-CoVs	1863	US	Respiratory, gastrointestinal	[24]
Cattle (*Bos taurus*)	BCoV	β-CoVs	1973	USA	Enteric, respiratory	[25]
Horse (*Equus caballus*)	ECoV	β-CoVs	2007	USA	Enteric	[26]
Human	229E	α-CoVs	1966	USA	Respiratory	[27]
	NL63	α-CoVs	2004	The Netherlands	Respiratory	[28]
	OC43	β-CoVs	1967	USA	Respiratory	[29]
	SARS-CoV	β-CoVs	2002	China	Respiratory	[30]
	HKU1	β-CoVs	2005	China	Respiratory	[31]
	MERS-CoV	β-CoVs	2012	Middle East	Respiratory	[32]
	SARS-CoV-2	β-CoVs	2019	China	Enteric	[33]

* IBV = infectious bronchitis virus, TCoV = turkey coronavirus, PhCoV = pheasant coronavirus, GfCoV = guinea fowl coronavirus (GfCoV), PiCoV = pigeon coronavirus, TGEV = transmissible gastroenteritis virus, PEDV = porcine epidemic diarrhea virus, PRCV = porcine respiratory coronavirus, SADS-CoV = swine acute diarrhea syndrome coronavirus, PHEV = porcine hemagglutinating encephalomyelitis virus, PDCoV = porcine deltacoronavirus, CCoV = canine coronavirus, CRCoV = canine respiratory coronavirus, FCoV = feline coronavirus, BCoV = bovine coronavirus, ECoV = equine coronavirus, SARS-CoV = severe acute respiratory syndrome coronavirus, MERS-CoV = Middle East respiratory syndrome coronavirus, SARS-CoV-2 = severe acute respiratory syndrome coronavirus 2.

**Table 2:** Diversity of coronavirus receptors.

Receptors	Virus *	Genus	Ref
Amino peptidase (APN, CD13)	Human CoV 229E	α-CoV	[66]
	TGEV		[67]
	PRCV		[68]
	FCoV		[69]
	CCoV		[70]
Angiotensin-converting enzyme 2 (ACE2)	Human CoV NL63	α-CoV	[71]
	SARS-CoV	β-CoV	[68]
	SARS-CoV-2	β-CoV	[72]
Dipeptidyl peptidase 4 (DPP4)	MERS-CoV	β-CoV	[73]
9-*O*-acetylated sialic acid	Human CoV HKU1	β-CoV	[74]
	PHEV	β-CoV	[75]
	Human CoV OC43	β-CoV	[76]
Carcinoembryonic antigen-related cell adhesion molecule 1 (CEACAM1)	MHV	β-CoV	[77]
Leukocyte antigen class I (HLA-1)	BCoV	β-CoV	[68]
	CRCoV	β-CoV	[72]
2,3-linked sialylated glycans	IBV	γ-CoV	[78]
Nonsialylated type 2 poly-LacNAc	TCoV	γ-CoV	[79]

* TGEV = transmissible gastroenteritis virus, PRCV = porcine respiratory coronavirus, FCoV = feline coronavirus, CCoV = canine coronavirus, human CoV NL63 = human coronavirus NL63, SARS-CoV = severe acute respiratory syndrome CoV, SARS-CoV-2 = severe acute respiratory syndrome coronavirus 2, MERS-CoV = Middle East respiratory syndrome coronavirus, human CoV HKU1 = human coronavirus HKU1, PHEV = porcine hemagglutinating encephalomyelitis virus, MHV = murine hepatitis virus, BCoV = bovine coronavirus, CRCoV = canine respiratory coronavirus, IBV = infectious bronchitis virus, TCoV = turkey coronavirus.

## Data Availability

Not applicable.
